# Production and Application of CAR T Cells: Current and Future Role of Europe

**DOI:** 10.3389/fmed.2021.713401

**Published:** 2021-08-16

**Authors:** Vladan Vucinic, Andrea Quaiser, Philipp Lückemeier, Stephan Fricke, Uwe Platzbecker, Ulrike Koehl

**Affiliations:** ^1^University of Leipzig, Medical Clinic for Hematology, Cell Therapy and Hemostaseology, Leipzig Medical Center, Leipzig, Germany; ^2^Fraunhofer Institute for Cell Therapy and Immunology, Leipzig, Germany; ^3^Department of Internal Medicine III Hematology, Oncology, Stem Cell Transplantation, Klinikum Chemnitz gGmbH, Chemnitz, Germany; ^4^Institute of Clinical Immunology, Medical Faculty, University of Leipzig, Leipzig, Germany; ^5^Institute for Cellular Therapeutics, Hannover Medical School, Hannover, Germany

**Keywords:** CAR T cells, cancer treatment, manufacturing, automation, regulation

## Abstract

Rapid developments in the field of CAR T cells offer important new opportunities while at the same time increasing numbers of patients pose major challenges. This review is summarizing on the one hand the state of the art in CAR T cell trials with a unique perspective on the role that Europe is playing. On the other hand, an overview of reproducible processing techniques is presented, from manual or semi-automated up to fully automated manufacturing of clinical-grade CAR T cells. Besides regulatory requirements, an outlook is given in the direction of digitally controlled automated manufacturing in order to lower cost and complexity and to address CAR T cell products for a greater number of patients and a variety of malignant diseases.

## Cell-Based Cancer Therapy: From Stem Cell Transplantation To Personalized Therapy With Car T Cells

The basis for cell-based cancer therapies was laid with the development of allogeneic hematopoietic stem cell transplantation (HSCT) in the 1960s (1)(1). From those beginnings to the present day, more than a million HSCTs have been performed around the world (2). After intensive conditioning therapy (chemotherapy or radiation), donor hematopoiesis is established as well as a graft-vs-tumor effect (3), as a result of which the donor's T lymphocytes recognize cancer cells as foreign and can kill them by various mechanisms. This effect was also described following the administration of donor lymphocyte infusions (DLI) for relapse treatment (4). However, differences in the HLA and/or minor histocompatibility antigens between donor and recipient can also trigger graft-vs-host disease (GvHD), which represents one of the most serious complications after allogeneic HSCT and can affect almost every organ system (5). In addition to identifying HLA-identical family donors, large registers are used to specifically search for HLA-compatible third-party donors. For many patients without HLA-compatible donors, haploidentical transplantation (donor and recipient share half of the HLA characteristics) is an established alternative. Despite a large number of foreign HLA antigens, T cell depleting drugs such as cyclophosphamide, applied immediately after transplantation, can reduce acute or chronic GvHD, with survival rates comparable to conventional HSCT (6, 7). The function and activity of the T cells in a hematopoietic cell transplant and the immune cells that develop after engraftment of the stem cells are therefore essential components of therapeutic success: an increase of the anti-tumor efficiency with simultaneous elimination or significant reduction of the T cell-mediated side effects (e.g., GvHD, cytokine release syndromes) is optimal. This has led to the development of the principle of modulating T cells as an essential part of immuno-oncological research and the generation of new therapeutic agents.

New antibody therapies are also making use of the impressive clinical potential of T lymphocytes. Checkpoint inhibitors such as ipilimumab, nivolumab, or pembrolizumab are monoclonal antibodies, the binding of which leads to the abolition of a mostly tumor-induced inhibition of T lymphocytes and thus to a therapy response (8). Furthermore, bispecific antibodies, which bind T cells in addition to the target antigen, are considered to be further developments of this principle (9). One example is blinatumomab, which is approved in the treatment of refractory or relapsed precursor B-cell lymphoblastic leukemia (r / r B-ALL) (10). This dual antibody fragment has binding sites both for CD19 (another antigen on B-ALL and B-lymphoma cells) and against CD3 (part of the T cell receptor) and thus leads to the formation of an immunological synapse between cancer cells and cytotoxic T lymphocytes (11).

In analogy to bispecific antibodies, certainly more complex reprogramming of T-lymphocytes can also be performed through transfer of the genetic information of an antibody-binding domain fused to essential T cell signaling domains, in context of therapy with CAR (chimeric antigen receptor) T cells. In this process, autologous T lymphocytes of the patient which recognize the target antigen are produced *ex vivo* through viral transduction of the CAR-T cells (12). These so-called living drugs were approved in USA 2017 and in EU 2018 are one of the most innovative therapy options for the treatment of aggressive B-cell lymphomas and the precursor B-ALL (<25 years).

**Figure 1:**
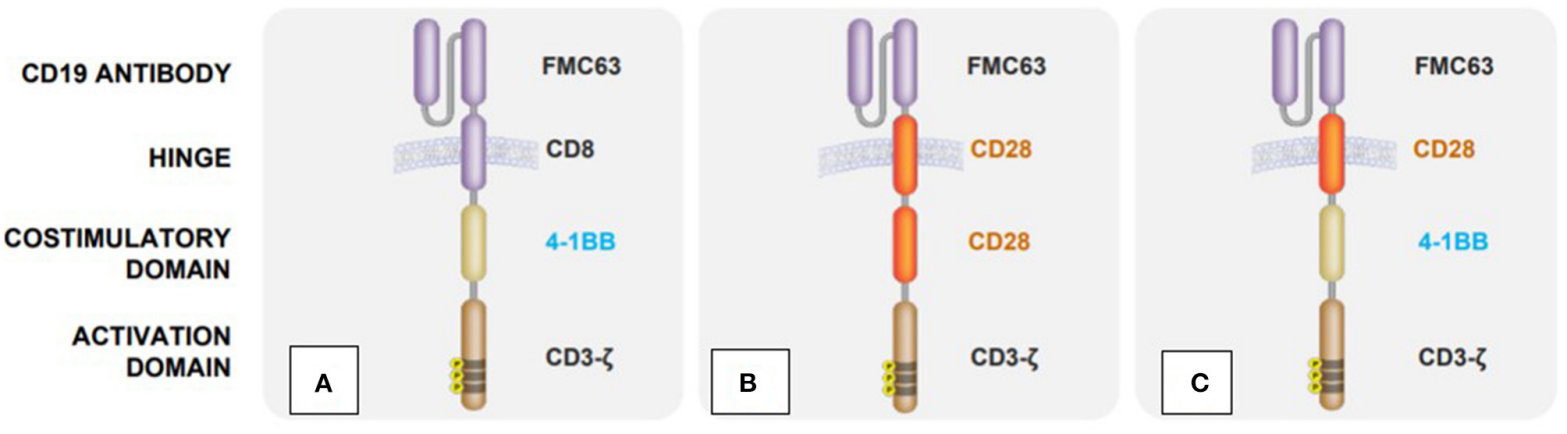
Structural differences between clinically available CAR T cell products: **(A)** Tisagenlecleucel (Kymriah) (14), **(B)** Axicabtagene ciloleucel (Yescarta) (15), **(C)** Lisocabtagene maraleucel (Breyanzi) (16).

After transduction, these cells express a variable domain of immunoglobulin, which, as an antigen receptor, is specifically directed against the surface antigens of cancer cells (13). Since these immunoglobulins are not physiologically expressed on T cells, these genetically modified T cells are also referred to as CAR T cells. Another difference to the natural T cell receptor is the fusion of costimulatory domains to the CAR molecule, which increase the efficacy of the cells (Figure 1) (17, 18).

Theoretically, it is possible to generate CAR T cells against a large number of relevant tumor antigens, nicely reviewed in (19–22). Once the tumor antigen has been recognized, the CAR T cells are activated, resulting in a targeted immune reaction directed against the respective tumor.

## Clinical Application Of Cd19-Car T Cells

One of the first clinical applications of CD19-CAR in hematology took place in 2009 in an intensively pretreated patient with follicular lymphoma (FL) where a partial remission was achieved by using CAR T cells (23). In 2010, the University of Pennsylvania started the first phase I study for adult patients with mature B-cell neoplasms (24). After the inclusion of three patients with chronic lymphatic leukemia (CLL), the study was stopped for financial reasons. An additional problem was the management of inflammatory reactions, summarized under the term cytokine release syndrome (CRS) (25). This may lead to life-threatening complications such as insufficient oxygen supply with the need for ventilation, severe hypotension with reduced blood flow to the periphery requiring circulatory support therapy, capillary leak syndrome with edema formation, especially of the lungs, but also multi-organ failure and disseminated intravascular coagulopathy (26). The publication of the positive clinical results of these patients (2 complete and 1 partial remission) lead to an increased global interest in CAR T cells (27). The goal of developing personalized immunotherapies and translating them into clinical application led to a cooperation between the University of Pennsylvania and Novartis in 2012, followed by a partnership between Kite Pharma and the National Cancer Institute (NCI) (28, 29). In 2013, the treatment results of two first pediatric patients with refractory or relapsed acute lymphoblastic leukemia (r/r ALL) were published (30). In addition, this was the first publication on the successful application of tocilizumab (anti-IL-6 antibody) in severe CRS. Further studies confirmed the surprisingly good complete remission rates in this patient cohort, which was previously considered as treatment refractory and thus incurable. (31). Additionally, the possibility of achieving a permanent remission for r/r ALL patients could be proven in a global multicenter study (25 centers in 11 countries) (32). The efficacy of CAR T cells has also been demonstrated in patients with lymphomas. The first phase II study was started at the University of Pennsylvania in 2014 in patients with r/r DLBCL and FL (33), followed by two multicenter international phase II studies for patients with refractory or recurrent diffuse large-cell B-cell lymphoma (r/r DLBCL) (34, 35). However, CAR T cell therapy may be associated with other complications in addition to CRS, such as immune effector cell-associated neurotoxicity syndrome (ICANS) and the macrophage activation syndrome. According to current recommendations from specific specialist societies, to treat CRS and to prevent this complication from progressing further, anti-IL-6 antibodies are given in its early stages (25, 36). For treatment of ICANS without CRS, corticosteroids are the therapy of choice. The standardized, stage-appropriate therapy of these possible complications requires the full-day availability of the anti-IL-6 antibodies in the clinic, as well as an interdisciplinary team for the immediate initiation of intensive medical, neurological and imaging measures, but also the continuous training of nursing and medical staff as summarized in the EBMT/ISCT recommendations (37, 38).

Three preparations are currently approved in the EU: tisagenlecleucel (Kymriah®) (39) and axicabetagene ciloleucel (Yescarta®) (40) for treatment of pediatric patients with r/r primary mediastinal B-cell lymphoma following at least two previous lines of therapy and brexucabtagene autoleucel (Tecartus®) (41) for treatment of mantle cell lymphoma in adult patients. EU approval for further drugs with other target antigens, e.g., B-cell maturation antigen, is expected in 2021.

So far, various pediatric and internal medicine centers have been certified for treatment with CAR T cell therapies that are associated with considerable additional logistical and infrastructural efforts. The number of centers varies in the individual EU countries depending on the organization of the health care system. There are only a few centers in centrally organized systems, whereas Germany with its decentralized, area-wide medical care concept has 26 centers (42). In Germany alone, CAR T cells for the treatment of patients with r/r CD19^+^ ALL/DLBCL are needed for approx. 1,200–1,400 patients per year (43).

## Car T Cells As Clinical Trial Products: Rules, Conditions, And Global Development

From a regulatory point of view, CAR T cells are an advanced therapy medicinal product (ATMP) in the EU. ATMPs are classified in (i) gene therapy medicinal products, including CAR T cells (ii) somatic cell therapy medicinal products, (iii) tissue-engineered products and (iv) combined ATMPs. They play a growing role in the treatment of cancer and hereditary diseases as well as in regenerative medicine and, more recently, in the development of therapies for viral infections. CAR T cells as an ATMP can be generated by either viral transduction leading to a permanent CAR expression or by using mRNA as well as transposon technology for transient CAR expression.

The manufacture, approval, and regulation of these innovative therapies are extremely complex and serve to protect the patients. They are subject to health and research policy framework as well as legal regulations that have a direct influence on international competitiveness. Therefore, the design of the framework is an important instrument to support research in the EU and to promote innovations. This, however, needs to be considered also in the context of international activities. The European Parliament and the Council of the European Union have issued Regulation (EG) No. 1394/2007 that regulates licensing, monitoring, and pharmacovigilance of ATMPs (44). Central approval is compulsory in the countries of the EU offering the advantage of market access in all EU member states. However, different regulatory frameworks within individual member states lead to complexity and reduce competitiveness. In Germany, for example, there are stricter regulations for the import of medicinal products and active ingredients from third countries than required by EU regulations (AMG § 72a). As a result of the lack of international harmonization in the recognition of certificates, manufacturers are obliged to carry out an acceptance inspection of the apheresis unit in non-EU countries. On the one hand, this obstacle affects the supply of CAR T cells for patients and, on the other hand, orders from abroad are lost, even if the manufacturer has a high level of professional qualification. The federal system that exists in Germany is also not conducive at this point. For example, the granting of a manufacturing license (AMG § 13) is subject to the respective state authority of the federal state and must be applied for a new in each other. Carrying out academically initiated studies requires considerable financial and human resources which University hospitals are currently unable to cover for the most part. Funding programs for such high financial volumes are only available to a limited extent. Due to the special nature of the production, there is an increased dependence on the industry. This situation prevents academic studies in Europe.

**Figure 2:**
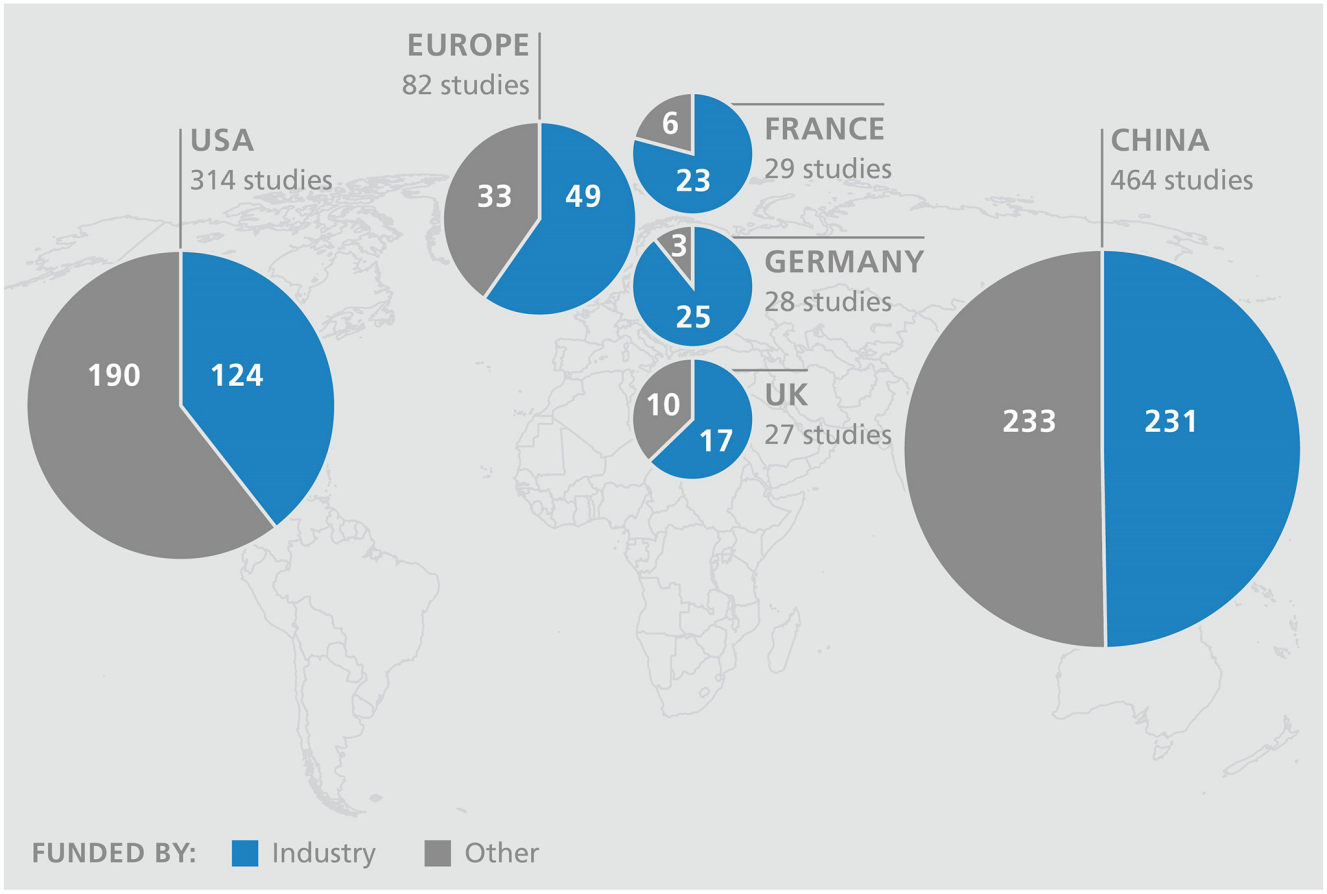
Top 5 countries of clinical CAR T cell studies with funding type (source: ClinicalTrials.gov).

Currently, there are around nine hundred studies worldwide with the use of CAR T cells as investigational drugs in different tumor entities, which confirms the increasing interest in immuno-oncology (45). There is good reason to hope that, in addition to addressing CD19+ hematological diseases, the development of therapies in oncology will also increase rapidly. The current Biotech Report of the Boston Consulting Group shows that only around 10% of studies are coordinated in Europe (46). Currently, China is the leader in this field, followed by the USA. Europe has already fallen behind its competitors and substantial investments & regulatory reforms are required to catch up. A look at the financing reveals a serious difference. While in Europe an average of 60 percent of the studies are sponsored by industry, the level is even significantly higher in some member countries (Germany 90%), in the USA and China more than half are initiated from the academic sector (Figure 2). In addition, a large amount of venture capital or governmental funding are available for the subsequent implementation in the USA and China - there are hardly any comparable options in Europe.

Furthermore, reliable reimbursement conditions and price setting must be put on the political agenda so that (i) patients can be guaranteed access to standard care with these high-priced therapies in the future (~1/4 million EUR/product for both approved CAR T cell products) without overwhelming the solidarity community and (ii) the financing gap in the clinics can be closed.

An innovative financing model was proposed by the pharmaceutical industry, which as so-called “Pay-for-Performance” provides for payments only if the treatment is successful (47). Manufacturing in strong networks using both centralized and decentralized manufacturing gives rise for future financial opportunities (48).

Government programs and financial support make a substantial contribution to support independent research and help to implement innovative ideas. In the field of cell and gene therapy, this could not only have a major influence on price formation of ATMPs, but would also strengthen the European position in this area. In its research and innovation investment program “Horizon Europe” approved at the beginning of the year, the European Commission provided a total budget of 95.5 billion euros, of this 25 billion earmarked for promoting scientific excellence.

## Technology Development And Transfer

So far, the focus has largely been on the development and improvement of the product, so the innovations are aimed at (i) CAR construct design up to the fourth generation, the TRUCK (T cells directed for antigen-unrestricted cytokine-initiated killing), (ii) non-viral vector formats like the Sleeping Beauty (SB) Transposon System and (iii) switchable universal CAR T platform technology (UniCAR), which allow to repeatedly turn the activity of CAR T cells on and off (49–51). However, the manufacturing process with its complex sequence of different process steps (1.) cell preparation, such as thawing and washing, (2.) selection, (3.) activation, (4.) transduction, (5.) expansion, (6.) harvest and (7.) final formulation of the cells, is still in an early development phase. Most of the products are manufactured under manual and only partially automated conditions.

After market approval of the first two CAR T cell preparations, the existing infrastructure is simply used for their production, which takes place in cooperation between the large pharmaceutical and biotechnology companies and their partners. In a second step, highly qualified specialist institutions are commissioned to guarantee the supply. In Germany, for example, there has been a collaboration between Novartis and the Fraunhofer Institute for Cell Therapy and Immunology (IZI) for the production of CAR T cells, initially as clinical test preparations, since 2015. So far, more than 500 CAR T cell preparations have been produced at IZI as part of this cooperation. There are currently about 150 qualified treatment centers in 20 countries in Europe including the UK, plus more than 200 outside the EU (52). The global supply of tisagenlecleucel (Kymriah®) is provided by seven manufacturers, 3 from Europe (France, Switzerland, Germany) and the others from the USA, China, Japan and Australia. In parallel, the same number of centers are qualified for the treatment with Yescarta^®^. To cope with the forecast, increase in the number of treatments, there is an urgent need for automation. Given the possibility to address not only hematological diseases, but also solid tumors, as a result, around 1.5 decimal powers more CAR T cell preparations must be made available. Upscaling is not a trivial process, but requires the optimization of each individual step and the analysis of the effects on the product by corresponding complex in-process and final product controls. In publications of some research groups, influences on the phenotype, exhaustion and senescence of the cells are described, which can lead to functional limitations (53, 54). Understanding molecular mechanisms is an important component in the development of new process strategies. An evaluation of clinical studies from the past 15 years reflects the diversity within the production of CAR T cells (55). This variability should be minimized to achieve a uniform robust process. In initial optimizations, open steps were replaced by closed steps in order to decimate the risk of product contamination. By reducing manual steps, which are extremely time-consuming and require the use of well-trained and highly qualified staff, the aim is now to increase the efficiency of implementation. The CliniMACS Prodigy^®^ from Miltenyi Biotec, for example, offers the possibility of decentralized production and is already being used as a proof of concept in several ongoing clinical trials for the production of CAR T cells (Table 1). This automatic and closed device is able to map all process steps from cell preparation to harvest.

**Table 1:** Automated CAR T cell production: Publications and clinical trials (14, 53, 54, 56–73).

**Author (year of publication)**	**Title**	**Device platform**	**Product/ Runs**
**a) Reviews**			
Fritsche E. et al. (73)	Toward an Optimized Process for Clinical Manufacturing of CAR-Treg Cell Therapy	1. GMP compliant cell sorter 2. Bioreactor 3. CliniMACS Prodigy^®^	
Mizukami A. and Swiech K. (72)	Platforms for Clinical-Grade CAR-T Cell Expansion Book: Chimeric Antigen Receptor T Cells (Chapter 10)	1. Bioreactor 2. CliniMACS Prodigy^®^ 3. Octane Cocoon^™^ cell culture system	
Smith D. et al. (70)	Toward Automated Manufacturing for Cell Therapies	1. Bioreactor 2. CliniMACS Prodigy^®^ 3. Octane Cocoon^™^ cell culture system	
Smith TA. (71)	CAR-T Cell Expansion in a Xuri Cell Expansion System W25 Book: Chimeric Antigen Receptor T Cells	Xuri Cell Expansion System W25	
Roddie C. et al. (69)	Manufacturing chimeric antigen receptor T cells: issues and challenges	1. Wave Bioreactor 2. G-Rex flask 3. CliniMACS Prodigy^®^	
Moutsatsou P. et al. (55)	Automation in cell and gene therapy manufacturing: from past to future	1. CliniMACS Prodigy^®^ 2. Octane Cocoon^™^ cell culture system 3. Quantum Cell Expansion (hollow fibers)	
Iyer R.K. et al. (74)	Industrializing Autologous Adoptive Immunotherapies: Manufacturing Advances and challenges	1. G-Rex static bioreactor 2. Wave-mixed Bioreactors 3. CliniMACS Prodigy^®^ 4. Octane Cocoon^™^ cell culture system 5. Quantum Cell Expansion (hollow fibers)	
Piscopo N.J. et al. (68)	Bioengineering Solutions for Manufacturing Challenges in CAR T Cells	1. Bioreactors 2. CliniMACS Prodigy^®^	
Kaiser A. et al. (67)	Toward a commercial process for the manufacture of genetically modified T cells for therapy	CliniMACS Prodigy^®^	
**b) Paper**			
Costariol E. et al. (66)	Demonstrating the Manufacture of Human CAR-T Cells in an Automated Stirred-Tank Bioreactor	Stirred tank bioreactor	CD19 CAR-T Donors (*n* =3)
Jackson Z. et al. (56)	Automated Manufacture of Autologous CD19 CAR-T Cells for Treatment of Non-Hodgkin Lymphoma	CliniMACS Prodigy^®^	CD19 CAR-T trial participants (*n* = 31)
Castella M. et al. (65)	Point-Of-Care CAR T-Cell Production (ARI-0001) Using a Closed Semi-automatic Bioreactor: Experience From an Academic Phase I Clinical Trial	CliniMACS Prodigy^®^	CD19 CAR-T trial participants (*n* = 28)
Fernández L. et al. (64)	GMP-Compliant Manufacturing of NKG2D CAR Memory T Cells Using CliniMACS Prodigy	CliniMACS Prodigy^®^	NKG2D CAR Memory T Cells validation runs (n = 4)
Vedvyas Y. et al. (63) Erratum in (2020)	Manufacturing and preclinical validation of CAR T cells targeting ICAM-1 for advanced thyroid cancer therapy	CliniMACS Prodigy^®^	ICAM-1 CAR-T preclinical validation (*n* = 7)
Aleksandrova K. et al. (53)	Functionality and Cell Senescence of CD4/CD8-Selected CD20 CAR T Cells Manufactured Using the Automated CliniMACS Prodigy^®^ Platform	CliniMACS Prodigy^®^	CD20 CAR-T establishing runs (*n* = 6)
Zhang W. et al. (62)	Characterization of clinical grade CD19 chimeric antigen receptor T cells produced using automated CliniMACS Prodigy system	CliniMACS Prodigy^®^	CD19 CAR-T establishing run (*n* = 1)
Blaeschke F. et al. (52)	Induction of a central memory and stem cell memory phenotype in functionally active CD4(+) and CD8(+) CAR T cells produced in an automated good manufacturing practice system for the treatment of CD19(+) acute lymphoblastic leukemia	CliniMACS Prodigy^®^	CD19 CAR-T autologous patients (*n* = 4)
Zhu F. et al. (61)	Closed-system manufacturing of CD19 and dual-targeted CD20/19 chimeric antigen receptor T cells using the CliniMACS Prodigy device at an academic Medical Center	CliniMACS Prodigy^®^	CD19 und CD20/CD19 CAR-T test runs (*n* = 7)
Lock D. et al. (60)	Automated Manufacturing of Potent CD20-Directed Chimeric Antigen Receptor T Cells for Clinical Use	CliniMACS Prodigy^®^	CD20 CAR-T test runs (*n* = 15)
Priesner C. et al. (59)	Automated Enrichment, Transduction, and Expansion of Clinical-Scale CD62L(+) T Cells for Manufacturing of Gene Therapy Medicinal Products	CliniMACS Prodigy^®^	GFP- T proof of principle *n* = 3 (4)
Mock U. et al. (58)	Automated manufacturing of chimeric antigen receptor T cells for adoptive immunotherapy using CliniMACS prodigy	CliniMACS Prodigy^®^	CD19 CAR-T test runs (*n* = 7)
**c) Clinical Trials**			
NCT04196413	GD2.BB.z.iCasp9-CAR T Cells	CliniMACS Prodigy^®^	*n* = 54
NCT03467256	CD19 CAR-T	CliniMACS Prodigy^®^	*n* = 18
NCT04049383	CAR-20/19-T cells	CliniMACS Prodigy^®^	*n* = 24
NCT03144583	CD19 CAR-T	CliniMACS Prodigy^®^	*n* = 28
NCT03434769	CD19 CAR-T	CliniMACS Prodigy^®^	*n* = 31
NCT03893019	CD20 CAR-T	CliniMACS Prodigy^®^	*n* = 15
Unknown	CD19 CAR-T	Cocoon^®^ Platform	*n* = 1

However, the small chamber volume, the insufficient flexibility and the restricted use, which occurs during the cell expansion phase of several days, have a limiting effect and could lead to a production bottleneck (56). In efforts to shorten the process, the cultivation time has been reduced from the usual 12 days to 8 days (57). Another automated system, the Cocoon^®^ platform from Lonza, was first used successfully last year at the Sheba Medical Center in Israel within a clinical trial (75). Alternatively, modular systems are used. Devices from various manufacturers, which only perform the respective process step automatically, are combined as needed. Widely used, even in commercial production, is the use of bioreactors for cell expansion. They can become a key element of industrialized manufacturing, as the new generation allows control of culture conditions and the possibility of process adaptation (74). The involvement of continuous monitoring of relevant process parameters and defined cell patterns would enable an adaptive process management. As an example, the Prodigy device is equipped with a microscope camera that already allows continuous monitoring of cell growth within the chamber. For the future, automated daily harvesting of small samples, which are transferred with a robotic arm into an external machine for cell characterization could improve early decision during a manufacturing process. Thus, the influence of subjective decisions and human-related protocol deviations could be minimized or eliminated (58). A modular system offers the decisive advantage of being able to organize the processes flexibly. The platforms are still not networked with one another in order to automatically map the entire process chain. The integration of different device and technology platforms for production and quality control in a digitally controlled process line would offer the flexibility and automation required for a large number of diverse cell and gene therapeutics and adaptations to further developments (56).

On the question of whether to favor centralized or decentralized manufacturing, existence of both is justified. In development and translation to the clinic, decentralized manufacturing in qualified GMP facilities of University hospitals plays an important role. The challenge in the commercialized manufacture of personalized therapies lies in the creation of various parallel independently running product manufacturing processes. This complexity calls for centralizing commercial production. Experience from other industrial sectors and the potential of the industry 4.0, characterized by the digitalization of production, can help to break new ground in the direction of robotic systems and intelligent automated process lines. Investments in the development of strategies for the automation and digitization of the production, product control and documentation of ATMPs must play a central role so that the global supply of patients with cell and gene therapeutics can be guaranteed in terms of availability of capacities, resources and finances.

## Conclusion

With its excellent and diverse research landscape, Europe still plays an important role worldwide. In contrast, more than 90 percent of clinical trials with CAR T cells are currently initiated outside Europe. Compared to the U.S. and China, venture capital funding is underdeveloped in Europe and regulations, decision processes and initiation of studies are lengthy and complex. The creation of appropriate framework conditions in an international context therefore seems essential to address and overcome (i) the delayed translation of research into the clinic, (ii) the lack of funding but also the increasing complexity of academically initiated phase I/II clinical trials, and (iii) improved support in the developments of automation and digitization of process routes in order to address 100-fold more patients moving from haematological to solid cancer. In the end, this will also determine how strongly Europe will be represented in the economic value added in the promising market of cell and gene therapy. Policymakers are therefore faced with the question of the extent to which they support science and create the conditions that are conducive to innovative developments in order to ultimately strengthen Europe as a location for research and business and not lose touch with the world leaders. Funding programs, such as Horizon Europe pave the way for better networking and cooperation among member states. However, efforts toward international harmonization of regulations must also be accelerated, because ultimately the huge challenges in the development and provide of personalized medicines cannot be met by national efforts alone, but only within the framework of international cooperation.

## Author Contributions

VV and AQ wrote the manuscript. PL, SF, UP, and UK provided administrational support. All authors listed approved the manuscript for the publication.

## Conflict of Interest

AQ and PL declare that the research was conducted in the absence of any commercial or financial relationships that could be construed as a potential conflict of interest. VV discloses honoraria for Novartis, Gilead, BMS, and travel grants from Gilead. SF states honorary activities for Novartis. With regard to the production of cell therapeutics, there are cooperations with the companies Novartis and Miltenyi Biotec. UP discloses consulting and fee for service activities for Novartis, Gilead and BMS. UK states that she is a consultant in immuno-oncology for AstraZeneca, Affimed, Glycostem, GammaDelta and Zelluna, and that she has collaborations with Novartis and Miltenyi Biotec regarding the production of CAR-T cells.

## Publisher's Note

All claims expressed in this article are solely those of the authors and do not necessarily represent those of their affiliated organizations, or those of the publisher, the editors and the reviewers. Any product that may be evaluated in this article, or claim that may be made by its manufacturer, is not guaranteed or endorsed by the publisher.
